# Sanitation of *Staphylococcus aureus* genotype B as a model for community of practice for improving food safety and animal health

**DOI:** 10.3389/fvets.2026.1835236

**Published:** 2026-06-09

**Authors:** Alicia Romanó, Hans Ulrich Graber, Diana R. Fonseca, Helena Stoffers, Noémie Matthey, Paula Teixeira, João Cortez, Maria Agustina Sarquis, Francois Bourdichon, Daniela Bassi, Carlotta Sartori, Luca Bacciarini, Michael Vaccani, Lorenzo Sesso, Susanne Lauber Fuerst, Patrizia Riva Scettrini, Martin Reist, Hans-Peter Bachmann, Fabian Wahl, Ghazal Nemati

**Affiliations:** 1 Agroscope, Food Microbial Systems, Bern, Switzerland; 2 CBQF - Centro de Biotecnologia e Química Fina– Laboratório Associado, Escola Superior de Biotecnologia, Universidade Católica Portuguesa, Porto, Portugal; 3 Department for Sustainable Food Process (DISTAS), Università Cattolica del Sacro Cuore, Cremona, Italy; 4 Repubblica e Cantone Ticino, Ufficio del Veterinario Cantonale, Bellinzona, Switzerland; 5 Innonext Sàrl, Brent, Switzerland; 6 Repubblica e Cantone Ticino, Ufficio della Consulenza Agricola, Bellinzona, Switzerland; 7 Federal Food Safety and Veterinary Office, Bern, Switzerland

**Keywords:** bovine mastitis, CATALYSE project, collaboration, community of practice, *S. aureus* GTB, sanitation program

## Abstract

Improving food safety and sustainability requires knowledge transfer from scientific studies to real-world applications, yet this process often faces significant barriers. This paper presents a seven-step model applied to a sanitation program that shows how focused scientific innovation can be successfully translated into practice to address complex food safety and animal health challenges. This model integrates active stakeholder engagement throughout all stages, from initial risk identification to pilot implementation. A cost-effective real-time quantitative PCR technique with sensitivity 99% and specificity 100% was used for the identification of *Staphylococcus aureus* genotype B (GTB)-infected herds (proof of concepts), enabling targeted control and intervention. The sanitation program demonstrated high efficacy, achieving a 73% reduction in *S. aureus* GTB-related mastitis cases within 7 months and complete eradication within 20 months across the 168 participating farms involving a total of 3,364 cows. The pilot phases of the program were expanded into a pragmatic implementation strategy developed by a consortium of researchers, veterinarians, farmers, and regulatory officials in Switzerland. Improved animal well-being, increased milk quality, farm productivity, and a notable decrease in antibiotic use for mastitis treatment were among the main benefits. Following the 2017–2020 implementation phase, a continuous monitoring program was established to sustain the project’s long-term success. We also discuss and evaluate the scalability of this local sanitation seven-step program initiative toward the establishment of an international community of practice within the EU-funded CATALYSE project (https://thecatalyseproject.eu/ and https://catalyse-cop.eu/). This case study validated a solid and cooperative roadmap for transforming fundamental scientific discoveries into viable, practical solutions. It emphasizes how an organized community of practice is essential to closing the gap between scientific advancement and end-user and can be adopted to facilitate scalability and replication around Europe, offering a useful model for enhancing food safety and sustainability throughout the larger dairy value chain.

## 1 Introduction

### 1.1 Background: barriers and prior work

Knowledge sharing is a key factor in improving food safety and sustainability, but it faces many barriers. Organizational silos and poor communication slow down the adoption of new practices (1), while fragmented data and weak interdisciplinary collaboration limit the flow of information across the food safety sector (2). To address these challenges, public–private partnerships and the application of technological innovations can strengthen the resilience of food systems by facilitating knowledge sharing (3, 4). However, significant challenges persist. Collaboration and information exchange among stakeholders—including industry, academia, and regulatory bodies—remain limited (3, 5). Regulatory and financial challenges are additional obstacles (6). For small and medium-sized enterprises, high implementation costs and limited access to finance reduce the capacity to implement new technologies (7, 8). Other reasons that limit the adoption of novel technologies include limited awareness of food safety innovations, unfamiliarity with innovative technologies among consumers and businesses/technologies, and information-access constraints faced by small and traditional producers (5).

Addressing these barriers requires targeted education and training initiatives to improve regulatory understanding and awareness of emerging innovations (4, 6). In this context, CATALYSE (“Catalyzing scientific innovation info food safety action,” project number 101136754) is a Horizon Europe Coordination and Support Actions project focused on accelerating and optimizing the uptake of food safety-enhancing knowledge and innovative solutions. The project focuses on building an online platform and an interactive community of practice (CoP) to facilitate connections among innovators, practitioners, regulators, and end-users through knowledge exchange.

### 1.2 Gap statement

The genotype-based diagnostic tools are a perfect example of a gap between the innovators and the end-users. Despite the existing frameworks providing the necessary structure, there is limited evidence on pragmatic, stakeholder-driven roadmaps that translate those tools into scalable sanitation programs.

This case study addresses this gap by presenting a practical model for applying basic scientific research in real-world settings.

### 1.3 Case study

The Swiss *Staphylococcus aureus* genotype B (*S. aureus* GTB) sanitation project served as a pilot example for the importance of the implementation of CATALYSE. In this project, scientific findings were successfully implemented due to the active involvement of researchers, authorities, and practitioners, who ensured the initiative met local needs, resulting in documented improvements in milk quality, animal health, and farm productivity.

The study also examined how EU-funded projects, such as CATALYSE, can support the expansion of such local innovations beyond national borders, since the project provides a structured and sustainable framework based on four actions “Collect–Translate–Facilitate–Educate” that help spread and adopt evidence-based solutions. The creation of a CoP connects end users from farms to forks in the food safety sector. This approach helps bridge gaps between stakeholders, align technological opportunities with real-world needs, and facilitate knowledge sharing across member states. In summary, this case study demonstrates how a local, field-tested innovation can expand to achieve a broader impact in Europe.

## 2 Context of the sanitation project

### 2.1 Challenges and needs of Swiss dairy farmers

Through sustainable and market-oriented production, Swiss agriculture and the dairy industry make an important contribution to ensuring a secure food supply (9). In 2023, Swiss agriculture produced marketable goods and services worth CHF 11.9 billion, with milk production accounting for 23.6% of this total (9). Summer pastures constitute an important source of income for Swiss farmers (10). The alpine season runs from late May to early October, during which around 25% of Switzerland’s dairy cows from different farms are brought together to spend the summer on pastures. Currently, around 6,000 summer pastures operate across multiple districts, including Bern, Grisons, Tessin, Valais, and others in central Switzerland (11).

**Figure 1:**
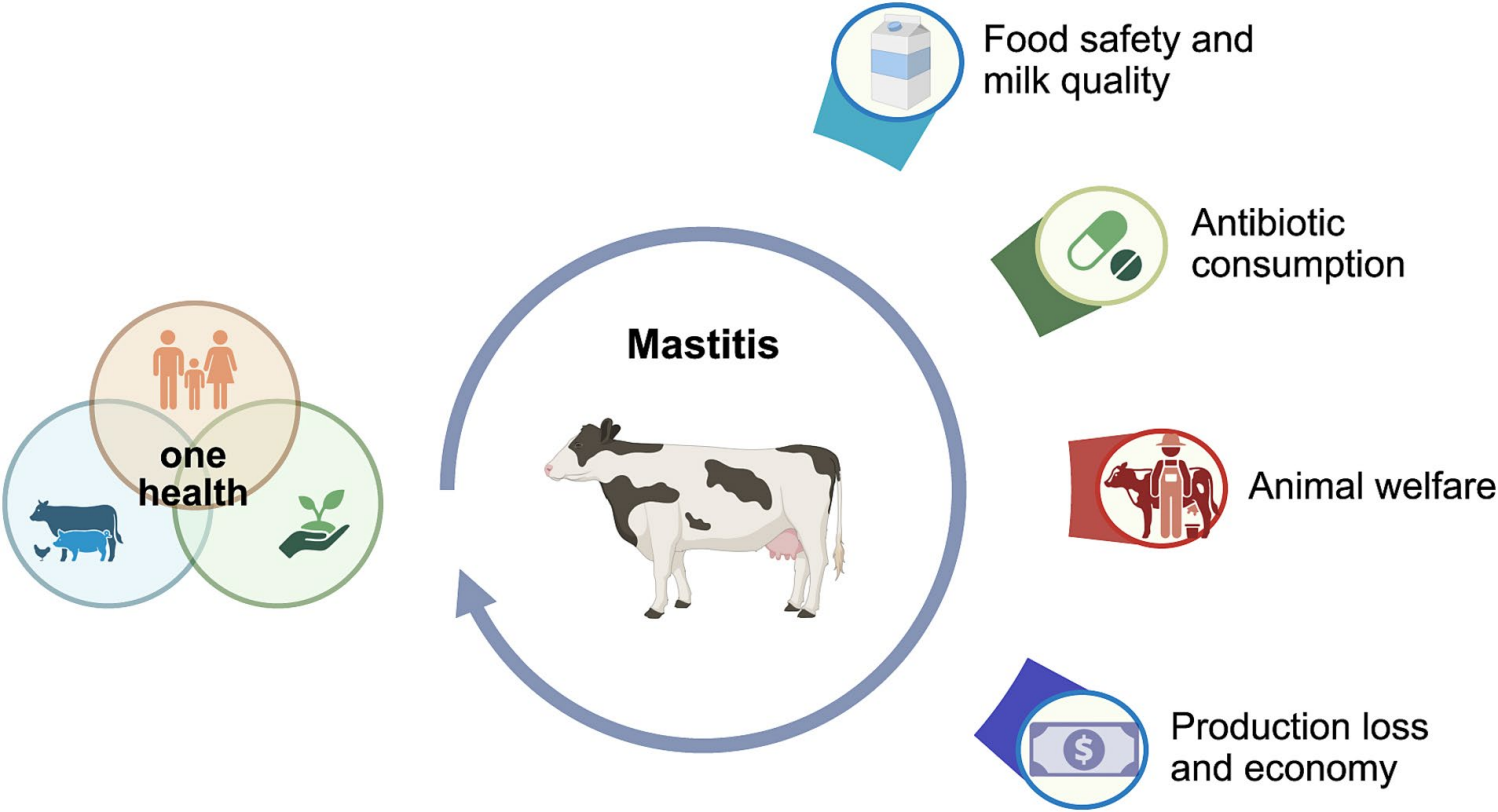
Schematic representation of challenges related to mastitis in the dairy sector. Created in BioRender. Romanó (2026): https://BioRender.com/c9mhjft and modified in Adobe Illustrator.

Bringing animals from different farms together on common summer pastures increases the risk of the transmission and spread of diseases such as mastitis. Bovine mastitis remains a major cause of economic loss in the dairy industry worldwide, due to reduced milk yield and quality, impaired cow health, and the rising costs of treatments and diagnostic tools required for disease detection (12) (Figure 1). According to a Swiss study, mastitis causes annual economic losses of approximately CHF 129.4 million in Switzerland, highlighting the substantial financial impact of this disease on the national dairy industry (13). *S. aureus* is one of the most widespread pathogenic microorganisms causing mastitis in dairy cows worldwide, including Switzerland (14, 15). Here, *S. aureus* GTB has been identified as a highly contagious subtype among all the genotypes detected (14). Due to its subclinical nature, this pathogen is considered a major contributor to the economic losses associated with mastitis in dairy herds (16, 17). Milk from infected cows contains high somatic cell counts (SCC), which leads to reduced milk quality and yield (18). These impacts are intensified by the contagious nature of the pathogen within herds. For example, the between-herd prevalence of *S. aureus* GTB on an alpine communal pasture increased from 27.3 to 56.6% by the end of the summer season, highlighting its rapid transmission potential (19).

In addition to its economic impact, *S. aureus* represents a public health problem due to its ability to produce heat-resistant enterotoxins that can contaminate raw and pasteurized dairy products (20). Enterotoxin production starts when bacterial counts in raw milk reach 10^5^ to 10^6^ colony-forming units (CFU)/g (21). Recently, Berger and coauthors showed the presence of *S. aureus* GTB in cheese sampled from the alpine regions of Austria, Italy, and Switzerland (22). Their study highlighted *S. aureus* GTB in milk as a key risk factor for the presence of staphylococcal enterotoxins in artisanal cheese products.

Control of *S. aureus* through conventional antibiotic therapy and vaccination has proven challenging and often unsatisfactory. Low cure rates for *S. aureus* intramammary infections have been widely reported (23–29), largely due to factors such as biofilm formation and the bacterium’s ability to colonize host cells, thereby evading antimicrobial agents (30). Although pathogenic bacteria causing udder infections generally showed low AMR, *S. aureus* might carry resistance to penicillin due to the inappropriate or inconsistent use of antibiotics (31–33).

### 2.2 Regulatory framework and strategic measures for mastitis control

The Swiss government and agricultural institutions have implemented various regulations and initiatives to manage and reduce the incidence of mastitis in dairy cows.

Total bacterial count is a key indicator of milk hygiene, with legal limits set by Regulation (EC) No 853/2004 varying by species and intended use: for raw cow’s milk the maximum is 100,000 CFU/mL, while for milk from other species it is 1,500,000 CFU/mL for bulk milk destined for heat treatment and 500,000 CFU/mL for raw milk sold directly for consumption (34); in Switzerland, a stricter limit of 80,000 CFU/mL applies to cow’s milk (35).

To ensure high-quality dairy products, Swiss regulations require strict hygiene practices in milk production. For instance, the “Guideline for the milk production and processing on alpine farms” (“Leitlinie für die gute Verfahrenspraxis bei der Milchgewinnung und -verarbeitung in Sömmerungsbetrieben”) provides recommendations for milk production and processing on alpine farms, emphasizing hygiene and quality control measures (36). Additionally, Switzerland enforces strict penalties for milk that exceed established quality standards for bacteria, SCC, and antibiotics. Dairies should produce milk with a bulk tank SCC of less than 350,000 cells/mL for three consecutive months to receive the full milk price, thereby incentivizing farmers to maintain low SCC levels and reduce mastitis incidence.

Regarding the use of antibiotics, a national system for reporting the use of antimicrobials at the farm level was introduced in Switzerland by the Federal Food Safety and Veterinary Office in 2019 based on a national strategy for decreasing antimicrobial resistance (37, 38). Since 2021, livestock farmers have been able to monitor their animals antibiotic use on their own farms and compare it with the prescriptions issued by their veterinarians. Since mid-March 2025, farmers have been able to compare their antibiotic use with that of other Swiss farmers, promoting self-monitoring as part of the Swiss Strategy on Antibiotic Resistance (38). In the EU, similar objectives are addressed in the Commission Notice on Prudent Use of Antimicrobials in Veterinary Medicine (2015/C 299/04) and Regulation (EU) 2019/6, which requires owners of food-producing animals to maintain detailed records of all pharmacotherapeutic treatments (Art. 108).

In the context of research and preventive initiatives aimed at reducing antibiotic use and, in general, mastitis, different projects have been launched in Switzerland and worldwide. For example, the Swiss project “ReLait” was launched to decrease the use of antibiotics at farms by implementing preventive measures to improve the health of calves, the uterus, and the udder (39). The results of the study showed that these preventive measures are effective in preventing mastitis (40). In Europe, from 2018, the “Nordic Mastitis Researchers’ Network” that includes Sweden, Norway, Finland, and Denmark provide guidelines on mastitis treatment with the goal to advisory and improving good practices in Northern farms (41). Internationally, organizations such as the National Mastitis Council, [10-point Mastitis Control Program, “Recommended Mastitis Control Program” (42)] and the International Dairy Federation are involved in providing best dairy practices and supporting farmers and veterinarians (43, 44). In addition, updated international reviews are available on general mastitis control strategies and, more specifically, on preventive measures for *S. aureus* (45, 46).

### 2.3 Geographical context of the project

**Figure 2:**
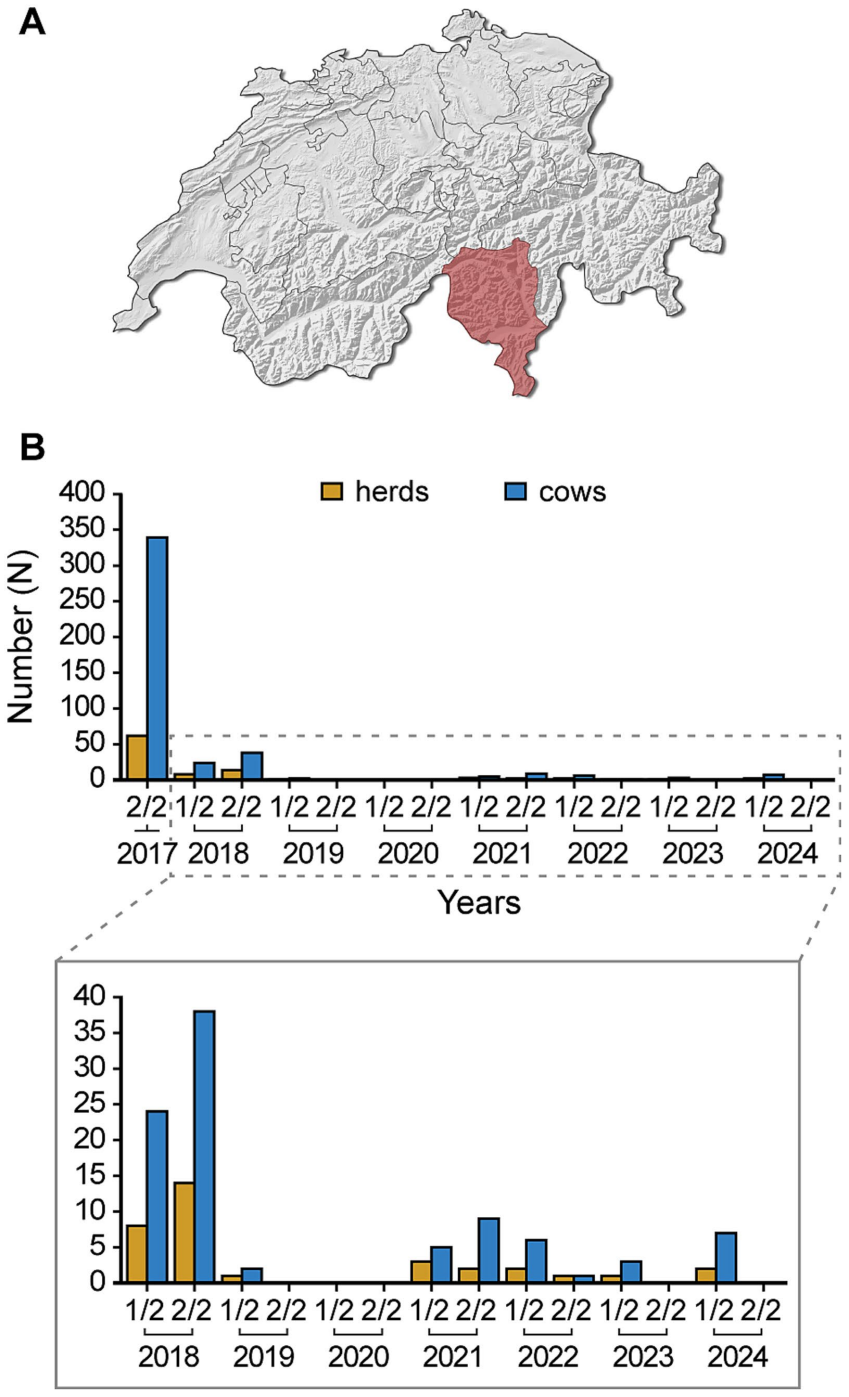
**(A)** The map of Switzerland highlights the Tessin region, where the sanitation program for *Staphylococcus aureus* GTB was performed. **(B)** Sanitation program objectives achieved. The *y*-axis represents the number (*N*) of the herds and/or cows positive to the *adlb* gene (GTB) based on qPCR detection; the yellow and blue columns represent the number of herds and cows positive to GTB, respectively. The *x*-axis represents the period of the year as 1/2, first 6 months and 2/2, the second part of the year. Map license *^©^ Agroscope* and refined in Adobe Illustrator. Graph generated from raw data and refined in Adobe Illustrator. Created in BioRender. Romanó (2026): https://BioRender.com/pffxluc and modified in Adobe Illustrator.

Since 2018, a sanitation program dealing with *S. aureus* GTB has been developed involving different Swiss institutions. This project was implemented as a pilot project in the Tessin region, an area where common alpine pasture during the summer months (May or June until mid-September) is a longstanding tradition (Figure 2A). Each summer, dairy cows from various farms in the district are brought together at different locations in the mountains (alps) for communal grazing and the production of alpine cow cheese. Due to the insufficient number of cows in the Tessin district to manage these pastures economically, additional animals are brought in from other Swiss districts (32). This unique management system significantly increases contact between animals from different herds, creating ideal conditions for the spread of contagious pathogens such as *S. aureus* GTB. Moreover, the Swiss dairy sector strongly supports the production of raw milk cheese, which depends on maintaining high milk quality. This presents additional concerns. The combination of high transmission risk due to communal pasturing and the potential impact on traditional dairy products makes the Tessin district a particularly relevant setting for implementing and evaluating targeted mastitis control measures (19, 47).

### 2.4 Organization and stakeholders involved

#### 2.4.1 Organizational setting

The sanitation program was developed and implemented under the scientific guidance of Agroscope, the Swiss center of excellence for agricultural research, which is affiliated with the Federal Office for Agriculture. Agroscope conducts research across the entire value chain of the agriculture and food sectors to support a competitive and multifunctional agricultural sector, high-quality food for a healthy diet, and an intact environment.

#### 2.4.2 Stakeholder involvement

The development and implementation of the program in Canton Tessin was made possible through the active involvement of a broad range of stakeholders at the federal, cantonal, institutional, and farm levels. The Swiss Federal Office for Agriculture and the Federal Food Safety and Veterinary Office played central strategic roles in founding the project, while the cantonal veterinary authorities ensured local coordination. Strong engagement from the Canton Tessin Milk Producers Federation, the Tessin farmers and their associations, together with the Swiss Milk Producers, facilitated the implementation of measures on farms. Local veterinarians acted as on-farm supervisors, translating recommendations into daily practice, while the Vetsuisse Faculty of the University of Bern contributed scientific expertise. Additional support from the Cantonal agricultural advisory office and professional agricultural organizations further enhanced knowledge transfer and farmer participation. This collaborative approach fostered shared ownership, strengthened the applicability of the program, and created a solid foundation for its implementation.

#### 2.4.3 Target population

The project was announced through information events for farmers and veterinarians, as well as the distribution of information materials, and dairy farmers in the canton of Tessin were encouraged to voluntarily participate in the program (32). A total of 168 dairy farmers (with 3,364 cows) in the Swiss canton of Tessin signed a contract to participate in the study and agreed to cooperate throughout the entire duration of the program from January 2018 to December 2020 (32).

### 2.5 Aims of the sanitation program

The *S. aureus* GTB sanitation program had several objectives. The primary goal was to reduce mastitis caused by *S. aureus* GTB. Additional aims included improving animal health and welfare, reducing the use of antibiotics for mastitis therapy, and improving the quality, quantity, and safety of milk and dairy products. Finally, the program was thought to evaluate its economic impact, with the expectation of increased income for farmers.

## 3 Details to understand key programmatic elements

A seven-step approach describes how Agroscope and its partners systematically identify, develop, and implement innovative solutions, starting with basic scientific research and a comprehensive understanding of farm-to-fork value chain. This summarizes all previous studies that were performed to develop the sanitation project. The proof of concept is reported in Sartori et al., whereas its application is described in detail by Sesso et al. (14, 32, 48).

The following subsections present and explain each of these seven steps in detail.

### 3.1 Basic research: radar screen phase—scanning the horizon for food safety hazards

Within 5 years of basic research from 2007 to 2012 in large animal medicine and working closely with dairy farmers to understand the real problems, Agroscope scientists identified that on the “radar screen,” infections with *S. aureus* were among the critical emerging hazards at the farm level (49–51). During the radar screen phase, stakeholders share observations of potential emerging food safety hazards. This process reveals the need for innovative solutions to address farm-to-fork food safety issues. A deep understanding of the entire value chain, which can only be achieved through highly interconnected networks and open sharing, is a prerequisite for developing the required insights and getting the “right things on the radar screen.”

### 3.2 Identify relevance—risk identification and characterization phase

Following the identification of the specific risks to dairy farmers and traditional cheese, a multidisciplinary research consortium, including the Swiss Federal Food Safety and Veterinary Authority, Agroscope, and the University of Bern, was founded to characterize the risk within the value chain and understand its significance for stakeholders. In a longitudinal study, the research consortium identified *S. aureus* GTB as the main strain contributing to contagious mastitis on small Swiss dairy farms. Previous studies showed that when *S. aureus* GTB was isolated, up to 87% of cows in the same herd were infected, confirming its high contagion rate and relevance to food safety (52). As an outcome, the study not only identified and characterized the risk but also confirmed the relevance of the problem and highlighted the need for innovative farm management practices to foster food safety and sustainability.

### 3.3 Develop innovative methodologies

After identifying *S. aureus* GTB as the main strain contributing to contagious mastitis, the research consortium recognized the need for an innovative detection method. To be applicable for sanitation programs on small dairy farms, such a solution had to be simple, inexpensive, and rapid so that farms could detect infected cases quickly. Consequently, Sartori and coauthors developed a real-time quantitative PCR (qPCR) assay for detecting the pathogen in milk samples, enabling identification in bulk tank milk samples containing at least one GTB-positive cow among 138 negative cows, with cow-level diagnostic sensitivity and specificity of 99 and 100%, respectively. This new assay specifically targets the contagiousness marker gene *adlb* (48).

### 3.4 Pilot experiment—connecting the dots

The innovative solutions identified through information sharing within the CoP were then translated into practice. Hence, the CoP can help transform theoretical knowledge into practice, with community experts facilitating information exchange and application by practitioners and end users. Formal or *ad hoc* working groups are organized and coordinated through the spiral operating network. To translate scientific knowledge into solutions that address the real needs of consumers, strong expertise was required to match the newly developed innovations with the needs of the volunteer pilot farms (21 herds involved). In this step, the team was expanded to include all relevant stakeholders, including pioneer farmers, to benefit from farm-to-fork expertise, to customize the tool to more practical and local needs, and to provide opportunities for training and education.

The pilot trial demonstrated that sanitation was successfully achieved using the newly developed qPCR approach. Key success factors included the simplicity of the method, which saved farmers time and money by enabling whole-herd sampling during regular milking procedures using their own trained farm staff. In addition, the high diagnostic sensitivity of the qPCR assay allowed for the accurate identification of *S. aureus* GTB-positive cows at any time point during lactation, allowing farmers to continuously update milking groups to prevent transmission. Through this innovation, milk sample analysis has become more precise, faster, less expensive, and more suitable for routine application, enabling the sanitation of even big herds within a short period (14).

### 3.5 Sanitation program in the Tessin region

The *S. aureus* GTB sanitation program was structured into three main stages:

- 2016–2017: Organizational preparation
- 2017–2020: Implementation of the sanitation program
- 2021–2024: Monitoring of the sanitation program

**Figure 3:**
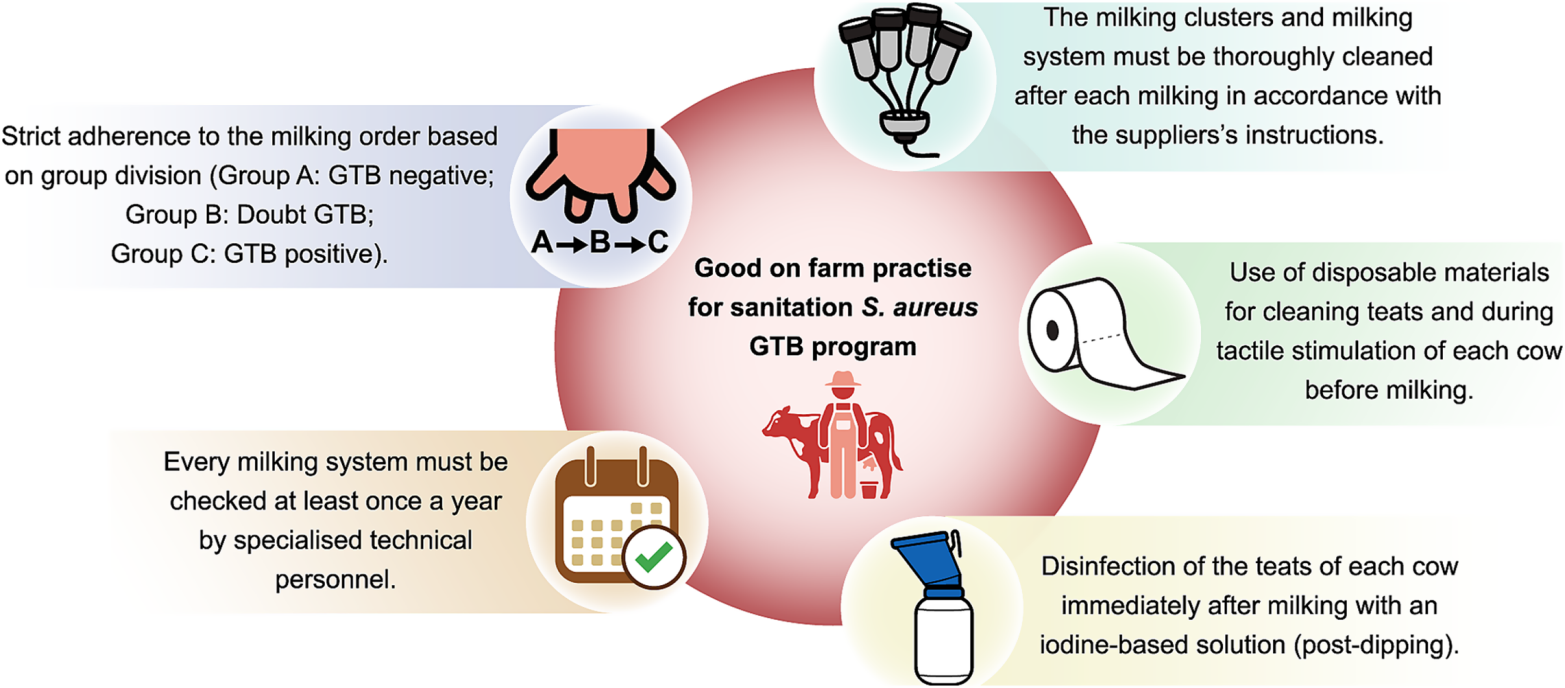
Five-point on-farm measurements involved in the *S. aureus* GTB sanitation program. Created in BioRender. Romanó (2026): https://BioRender.com/pffxluc and modified in Adobe Illustrator.

For organizational preparation, farmers were provided with an informed consent form for participation in the sanitation project and were required to sign it. Participation was voluntary and required at least 75% approval from farmers to start. During the program, it was necessary to increase several critical phases to ensure its effectiveness. The purpose of this step of the project was to understand the validation of the pooled sample (10 cows, 1 mL/cow), design a decision-making protocol in the case of positive cases (culling or therapy), identify on-farm measurement techniques, validate hygiene practices and milking procedures based on health status (Figure 3), and provide alpine seasonal management recommendations. Furthermore, implementation and monitoring programs were developed to assess the *S. aureus* GTB status of animals before and after entry into the alpine region. In cases where animals were culled, financial compensation was provided.

The project achieved a 73% reduction in *S. aureus* GTB-infected herds within 7 months of beginning the sanitation program; all herds were *S. aureus* GTB-free after 20 months (at the cow level: twice qPCR negative in a row; at the farm level: all cows of a herd were free from *S. aureus* GTB) (Figure 2B). Alongside the decline in mastitis, milk quality improved, resulting in higher prices, and food safety was enhanced (32). Animal welfare also benefited, with less painful mastitis, reduced animal wear, and lower rearing costs due to milking cows over more lactations. Socially, farmer stress decreased as managing contagious herd problems became easier. Finally, the use of antibiotics for mastitis therapy on Alps was significantly reduced (53).

### 3.6 International research and evaluation of the needs for knowledge transfer for small-scale dairy farmers in Europe

Several studies have reported that *S. aureus* genotypes differ in their ability to spread within a herd. *S. aureus* GTB/CC8 *adlb*-positive was associated with a high prevalence of intramammary infection within the herd; the *adlb* gene appears to be specifically linked to *S. aureus* GTB/CC8 and could be a potential marker of contagiousness. By contrast, other genotypes are generally associated with disease in individual cows (54, 55). Previous research has investigated the diversity of genotypes across European and international countries (55, 56) and the presence of the *adlb* gene in some affected herds in those countries (48). In 2016, Cosandey and coauthors analyzed strains from 12 European countries and identified five major genotype clusters: CLB, CLC, CLF, CLI, and CLR. In particular, GTB was isolated in strains from central Europe. These same strains were additionally screened for the presence of the *adlb* gene (31, 55), revealing a statistically significant association between GTB and the presence of the *adlb* gene.

In Germany, mastitis strains, *adlb* was also associated with other genotypes, such as genotype R (31). Monistero and colleagues reported that GTB was isolated only from *S. aureus* isolates from Italy but not in other countries, suggesting the need to develop specific approaches based on the major genotype detected (56). In another study performed by Maisano and colleagues, 60 dairy herds in northern Italy showed the crucial role of the genetic properties of *S. aureus*, especially the role of the *adlb* gene, which could be related to determining the prevalence of intramammary infections within a herd (57). This implies that the risk observed is also relevant for other countries in the European food system and that the innovative solution, both the technology and the education and training programs, can be easily transferred and adapted to other European dairy farms, depending on their condition, size, and needs.

### 3.7 Adapt and roll out innovation from Switzerland to EU/transition countries through EU food safety network

**Figure 4:**
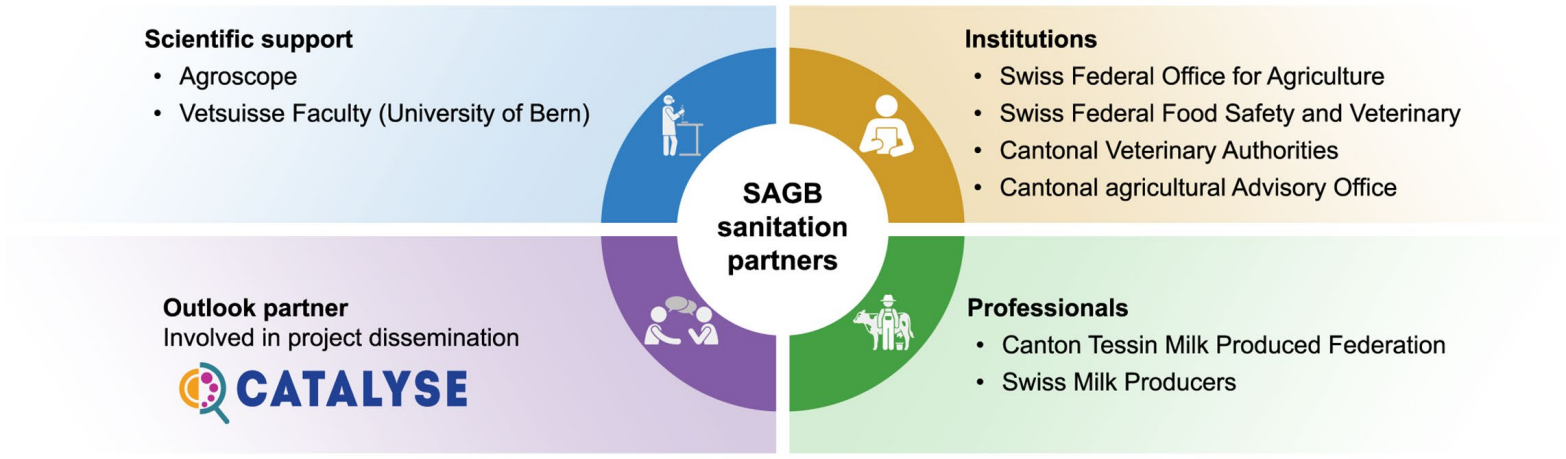
Stakeholders involved in the sanitation program and their role in the project. SAGB: *S. aureus* genotype B. Created in BioRender. Romanó (2026): https://BioRender.com/4a846d4 and modified in Adobe Illustrator.

In the sanitation project, the key to success was finding the right partners and actors in Switzerland (Figure 4). Extending the project to other European countries requires the same approach. There is an urgent need for a platform that enables exchanges among all food safety stakeholders across the entire value chain, from farmers to consumers, to pave the way for innovations and accelerate their dissemination. Such a platform would catalyze the communication process, provide access to key solutions, highlight emerging food safety issues, connect the dots, and introduce scientific innovation at the right place and time. The initiative needs the support of a structured framework and a data center to provide stakeholders at all levels with access to relevant information.

## 4 Discussion

The Agroscope case study presents a structured approach designed to support the successful establishment of a CoP in food safety innovations, namely in the dairy sector. Based on the “Collect–Translate–Facilitate–Educate” CATALYSE working model, this case study followed a seven-step process to transform research into practical innovation sharing. This process begins with comprehensive research and hazard identification, proceeds with the development of targeted tools for risk assessment, and culminates in pilot studies that validate the feasibility and economic benefits of the proposed solutions.

The initial phase consisted of identifying a practical challenge (mastitis in dairy) and designing and creating research programs to address it (e.g., a mastitis sanitation program). The correct selection of the beneficiary and stakeholders directly influences the success of testing the idea and innovations with the end users (e.g., local/small farmers). Effective, active, and reciprocal communication among all involved stakeholders is essential to correctly translate theoretical knowledge into practical solutions. Moreover, this model reinforced the importance of iterative testing and refinement, as well as the importance of structured feedback to ensure solutions that are not only scientifically robust but also practical, economically viable, and aligned with end users needs (58–60).

*S. aureus* GTB was identified as the main strain responsible for contagious mastitis on small Swiss dairy farms, associated with high treatment and management costs. After risk identification, early stakeholder engagement and the involvement of external experts play a crucial role in ensuring the relevance of this approach and potential solutions (58). The participation of authorities, such as the Swiss Federal Food Safety and Veterinary Authority, along with researchers and industry, contributes to a comprehensive and multidisciplinary risk assessment. A commonly reported reason for CoP failure is the lack of clear stakeholder identification and engagement in the development and follow-up of shared materials and resources (58). This case study demonstrates how theoretical research can be effectively translated into practical tools. The development of a qPCR assay for detecting *S. aureus* GTB in milk provides a user-friendly diagnostic solution for dairy farmers to identify GTB-positive cows. The integration of the qPCR assay into systematic mastitis sanitation programs enabled early detection and more targeted interventions, supporting improved animal health, farm productivity, and sustainability. Additionally, from an economic perspective, the costs of screening and diagnosis increased at the cantonal level. However, positive improvements were observed at both the animal and farm levels, and epidemiological results demonstrated that the control program was highly effective (61).

From a methodological perspective, mastitis is a multifactorial disease, not limited to *S. aureus*. Therefore, this method is most applicable in regions where *S. aureus* GTB mastitis is a primary concern. Based on Sartori et al. and Boss et al., the GTB assay in bulk tank milk is limited due to differences in bacterial shedding and is not reliable based on a single measurement (48, 50). In contrast, at the cow level, two negative PCR tests provide high diagnostic sensitivity (approximately 99% detection). This can be a limitation for the method applicability to different regions. Additionally, stakeholders, their language, and geographical context play an important role. Successful implementation depends on effective communication in a clear and understandable language, while benefiting from strong connections with local stakeholders such as farmer unions, veterinarians, and advisory bodies. Regional conditions, including climate and farming practices, must also be considered, aligned with the regional policy framework, including incentives, milk quality regulations, and antibiotic use policies. In addition, the quality of milk data collection, such as somatic cell count and recording systems, has a major impact on the reliability of the model. Nevertheless, the model represents a valuable pilot approach, demonstrating how a comprehensive, preventive hygiene system in dairy production can be developed through systematic collaboration.

A successful CoP encompasses defined strategic objectives, a clear strategic implementation plan, and effective communication channels between solution providers and end users. The core team that forms a CoP between the university, public authorities, and end users is essential to operate in a complex matrix of science, regulation, and economics. Those parameters should be aligned with strong leadership, and the effective use of digital platforms for knowledge sharing and collaborative learning (58, 60, 62, 63). The Agroscope model aligns with these principles, offering a structured roadmap for the dissemination, scaling up, and adaptation of innovations across different contexts. For instance, studies on CoP models emphasize the importance of establishing a dedicated core group and implementing structured feedback mechanisms to facilitate the sharing of best practices (63). These insights align with the Agroscope approach, which prioritizes early stakeholder engagement and iterative risk assessments to translate research into practical applications.

The integration of technology and digital tools further enhances the effectiveness and range of a CoP. The CATALYSE project will promote knowledge sharing and exchange through a structured online platform. Studies of other CoPs indicate that incorporating webinars, interactive discussions, and mentorship programs significantly improves member engagement and knowledge retention. These digital resources create an environment in which continuous learning and adaptation are encouraged, fostering long-term sustainability (64, 65). Continuous training and feedback expand the circle of users of the program, thereby increasing its viability and improvement. Digital platforms in global networks were fundamental in facilitating rapid knowledge exchange and real-time updates, resource sharing, and collaborative problem-solving.

Moreover, long-term commitment and structured governance are crucial for maintaining a CoP relevance and effectiveness over time. The Agroscope case study illustrates these principles in practice through its expansion beyond Switzerland and the EU, demonstrating how a well-organized CoP can extend its impact on sustainability and the reduction of antibiotics consumption. Similar case studies highlight that sustained engagement, strong leadership, and dedicated facilitation are key to driving continuous innovation (60, 62, 64, 66).

### 4.1 Synergies between the Agroscope case study and the CATALYSE community of practice

Based on observations within the Swiss dairy community, Agroscope developed a basic research program on mastitis sanitation to help small-scale farm economics, foster animal health, ensure the sustainability and long-term business viability of local cheese production, and improve food safety. Agroscope followed a seven-step approach to identify, develop, and launch innovative solutions to reach a community of end-users, starting from basic science and a deep understanding of the farm-to-fork value chains (32). Briefly, it began with horizon scanning for food safety hazards and risk identification, revealing *S. aureus* GTB as a key mastitis agent in Swiss dairy farms. A simple and cost-effective qPCR diagnostic tool was developed and successfully implemented in pilot sanitation programs, demonstrating health, economic, and sustainability benefits aligned with a reduction in antibiotic consumption. Expanded to the European level, the prevalence of mastitis caused by *S. aureus* was investigated in 10 countries, showing clear relevance. The method employed by local commercial laboratories as a diagnostic tool and has proven to be successfully applicable. Based on Sesso et al., it was shown to be applicable across different Swiss farming systems (32). Furthermore, the application of the method has already been established in public veterinary diagnostic institutions in Italy for screening purposes. To further improve applicability, the model should be expanded beyond *S. aureus* to include other major mastitis pathogens, as well as environmental and management-related risk factors.

This case study demonstrates the critical importance of effective communication and the translation of scientific knowledge into a shared, accessible language that can be understood and adopted by different stakeholders across different regions and countries. Addressing these challenges requires not only strong scientific approaches but also innovative, easy-to-use solutions that can be rapidly adopted and disseminated through targeted training programs.

The CATALYSE CoP^1^ was envisioned as an online platform to facilitate the translation of innovation to foster knowledge exchange between end users and connect R&D ecosystems. More than a knowledge and theoretical hub, the CoP serves as a platform for collaborative discussion where shared challenges can be addressed collectively. The “Collect–Translate–Facilitate–Educate” model promotes an environment of fast problem-solving and continuous improvement, due to the different members’ perspectives and expertise. The information centralized in one online platform enabled stakeholders from different countries to access, exchange, and adapt solutions in a coordinated and efficient manner. The CoP will be a vehicle for:

- Early screening to detect and anticipate risks and issues in the food system.
- Risk discussion and documentation collection through forums and sharing spaces.
- Knowledge repository that collects, selects, and makes knowledge about practical innovative solutions available or helps to understand whether similar methodologies can be adapted.
- Knowledge translation to all stakeholders who can share experiences, support training and education, and implement innovative solutions.
- Scalability for other European end users.
- Collaboration and information-sharing processes, connection of value chain actors, and access to solutions.

This case study demonstrated the urgency of creating a platform that enables exchanges between all food safety stakeholders involved in the entire value chain, from farmers to consumers, paving the way for innovations. A platform that catalyzes the communication process, provides access to key solutions, and highlights emerging food safety issues to connect the dots and introduce scientific innovation at the right place and time. The initiative will be supported in the form of a structure and data center to provide access to all levels of stakeholders.

The Agroscope case study provides a replicable and reproducible roadmap for achieving this vision, demonstrating how research-driven collaborative frameworks can drive sustainable transformation in the food safety sector.

## Data Availability

The original contributions presented in the study are included in the article/supplementary material, further inquiries can be directed to the corresponding author.

## Footnotes

1
https://catalyse-cop.eu/
